# Deep Learning and Automatic Differentiation of Pancreatic Lesions in Endoscopic Ultrasound: A Transatlantic Study

**DOI:** 10.14309/ctg.0000000000000771

**Published:** 2024-09-26

**Authors:** Miguel Mascarenhas Saraiva, Mariano González-Haba, Jessica Widmer, Francisco Mendes, Tamas Gonda, Belen Agudo, Tiago Ribeiro, António Costa, Yousef Fazel, Marcos Eduardo Lera, Eduardo Horneaux de Moura, Matheus Ferreira de Carvalho, Alexandre Bestetti, João Afonso, Miguel Martins, Maria João Almeida, Filipe Vilas-Boas, Pedro Moutinho-Ribeiro, Susana Lopes, Joana Fernandes, João Ferreira, Guilherme Macedo

**Affiliations:** 1 Department of Gastroenterology, Precision Medicine Unit, São João University Hospital, Porto, Portugal;; 2 WGO Gastroenterology and Hepatology Training Center, Porto, Portugal;; 3 Faculty of Medicine of the University of Porto, Porto, Portugal;; 4 Hospital Universitario Puerta de Hierro Majadahonda, Madrid, Spain;; 5 New York University Langone Hospital, New York, New York, USA;; 6 New York University Manhattan Hospital, New York, New York, USA;; 7 Hospital Das Clinicas da Faculdade de Medicina da Universidade de São Paulo, São Paulo, Brazil;; 8 Faculty of Engineering of the University of Porto, Porto, Portugal.

**Keywords:** artificial intelligence, cystic pancreatic lesions, deep learning, endoscopic ultrasound, solid pancreatic lesions

## Abstract

**INTRODUCTION:**

Endoscopic ultrasound (EUS) allows for characterization and biopsy of pancreatic lesions. Pancreatic cystic neoplasms (PCN) include mucinous (M-PCN) and nonmucinous lesions (NM-PCN). Pancreatic ductal adenocarcinoma (P-DAC) is the commonest pancreatic solid lesion (PSL), followed by pancreatic neuroendocrine tumor (P-NET). Although EUS is preferred for pancreatic lesion evaluation, its diagnostic accuracy is suboptimal. This multicentric study aims to develop a convolutional neural network (CNN) for detecting and distinguishing PCN (namely M-PCN and NM-PCN) and PSL (particularly P-DAC and P-NET).

**METHODS:**

A CNN was developed with 378 EUS examinations from 4 international reference centers (Centro Hospitalar Universitário São João, Hospital Universitario Puerta de Hierro Majadahonda, New York University Hospitals, Hospital das Clínicas Faculdade de Medicina da Universidade de São Paulo). About 126.000 images were obtained—19.528 M-PCN, 8.175 NM-PCN, 64.286 P-DAC, 29.153 P-NET, and 4.858 normal pancreas images. A trinary CNN differentiated normal pancreas tissue from M-PCN and NM-PCN. A binary CNN distinguished P-DAC from P-NET. The total data set was divided into a training and testing data set (used for model's evaluation) in a 90/10% ratio. The model was evaluated through its sensitivity, specificity, positive and negative predictive values, and accuracy.

**RESULTS:**

The CNN had 99.1% accuracy for identifying normal pancreatic tissue, 99.0% and 99.8% for M-PCN and NM-PCN, respectively. P-DAC and P-NET were distinguished with 94.0% accuracy.

**DISCUSSION:**

Our group developed the first worldwide CNN capable of detecting and differentiating the commonest PCN and PSL in EUS images, using examinations from 4 centers in 2 continents, minimizing the impact of the demographic bias. Larger multicentric studies are needed for technology implementation.

## INTRODUCTION

Endoscopic ultrasound (EUS) allows for an adequate evaluation of pancreatic lesions, being the preferred modality for tissue sampling of focal pancreatic lesions (1). The diagnosis of focal pancreatic lesions is challenging. These lesions can manifest as solid, cystic, or mixed tumors and have either a benign, precancerous, or malignant histology (2).

Pancreatic cystic neoplasms (PCN) are commonly an incidental finding in imaging examinations, with an estimated prevalence of 8% in a recent meta-analysis (3). The detection of a PCN is associated with a 19-fold increased risk of pancreatic adenocarcinoma (4). However, malignancy risk is very different according to PCN type (5). PCN can have a myriad of causes, namely congenital, inflammatory, or neoplastic. Nevertheless, the malignancy risk is almost exclusive of PCN with a mucinous phenotype (M-PCN). The commonest M-PCN is intraductal papillary mucinous neoplasm, with indication for surgery in lesions with clinical or imaging worrisome features (6).

Pancreatic solid lesions (PSL) are commonly diagnosed as incidentalomas, with a prevalence of around 0.5% in computed tomography studies of asymptomatic individuals (7,8). The differential diagnosis contemplates pancreatic ductal adenocarcinoma (P-DAC), pancreatic neuroendocrine tumors (P-NET), and other rarer causes. P-DAC is the commonest cause of PSL. Indeed, P-DAC has a poor prognosis, with a 5-year survival rate of 2%–3% because of disease diagnosis with advanced irresectable disease stages (9). On the other hand, P-NETs are less frequent neoplasms with malignant potential. Surgical therapy is commonly curative, but conservative management is adequate for small low-grade nonfunctioning P-NETs (10).

EUS is useful for identification of PCN with criteria for surgical resection. However, its diagnostic yield is modest. In fact, EUS has a relatively low accuracy for differentiating M-PCN from nonmucinous PCN (NM-PCN), with an accuracy ranging from 48% to 94% between studies (11). In addition, the lack of interobserver agreement is a problem in EUS, with low or modest interobserver agreement in the evaluation of PCN (12).

EUS is also considered the imaging method of choice to exclude P-DAC, with 94% sensitivity for detection of PSL (13,14). Moreover, EUS is also superior to conventional imaging modalities for diagnosing P-NETs, being the best modality for diagnosis of lesions under 10 mm, commonly overlooked by computed tomography (15). In addition, EUS is capable of assuring characterization and tissue sampling of pancreatic lesions. In fact, EUS-guided tissue sampling can be achieved through fine needle aspiration (FNA) or biopsy (FNB) (16). A recent systematic review with metanalysis showed that EUS-FNB had better diagnostic accuracy than EUS-FNA for P-DAC (17). Nevertheless, EUS-FNB has diagnostic accuracy of 91% for P-DAC under 10 mm, with a non-neglectable risk of missing malign cases (18). Moreover, the diagnosis of P-DAC is especially difficult in the presence of chronic pancreatitis (19). This creates the need for additional tools to augment the diagnostic accuracy of EUS-guided biopsy.

The development and implementation of artificial intelligence (AI) algorithms, namely deep learning models, have been focus of interest in diverse medical areas (20,21). Gastroenterology, as a specialty with a strong image component, has been the focus of studies with promising results. Convolutional neural networks (CNN) are type of multilayer human visual cortex-inspired algorithm, with high accuracy for image analysis (22). Despite the potential benefit of deep learning algorithms in EUS, there are only a few works about the role of CNN in EUS for pancreatic lesions (23,24). With this study, our group aimed to develop the first worldwide CNN for automatic diagnosis and differentiation of both PCN and PSL in EUS images.

## METHODS

### Study design

Our group developed a retrospective multicenter study based on patients submitted to EUS for pancreatic lesion characterization. A video file was created for every patient whose EUS examination was recorded. The EUS examinations were performed with 3 different linear EUS devices: Olympus GF-UCT180, Olympus GF-UC140 echoendoscope, and SonoScape EG-UC5T echoendoscope. Images obtained from each examination were used for the development, training, and validation of a CNN model for automatic detection and differentiation of both PSL and PCN.

This study was approved by the ethics committee of Centro Hospitalar Universitario São João/Faculty of Medicine of the University of Porto (Comissão de Ética 41/2021), Hospital Universitario Puerta de Hierro Majadahonda (PI 153/2022), New York University Langone and Manhattan Hospital (Institutional review board 03845978/2023), and Hospital das Clínicas da Faculdade de Medicina da Universidade de São Paulo. This study was developed in a noninterventional nature, and the patient management was not affected by study design our results. A team with Data Protection Officer certification assured the nontraceability of data according to the general data protection regulation. Therefore, data anonymization was achieved through assignment of a random number to each patient.

### EUS procedure and definitions

EUS procedures were performed under anesthesiologist-directed sedation using 3 different linear echoendoscopes: Olympus GF-UCT180 (Olympus EU-ME2 ultrasound processor), Olympus GF-UC140 (Olympus EU-ME2 ultrasound processor), and SonoScape EG-UC5T (SonoScape S60 Ultrasound System). EUS procedures were performed by endoscopists with more than 250 EUS examinations (F.V.B., S.L., P.M.R., M.G., B.C., J.W., and M.S.).

The histopathological diagnosis for PSL was achieved either through EUS-guided tissue sampling (FNA or FNB) from the primary tumor or a metastatic tumor. In addition, the diagnosis of a PSL could be based on a percutaneous biopsy, surgical biopsy, or histopathological analysis of the surgical sample. When performing EUS, the use of FNB vs FNA and the number of performed biopsies was a decision of the expert endoscopist. Regarding P-NET, lesions with a cystic component were included in the P-NET group because of the confirmation of the histopathology analysis either through percutaneous biopsy or surgical sampling.

On the other hand, when considering PCN, the division between NM-PCN and M-PCN was based on histopathological analysis of EUS-FNA or fluid analysis (with measure of glucose and carcinoembryonic antigen levels). Alternatively, the diagnosis could be achieved through the analysis of the surgical sample. PCN were considered mucinous in the presence of cytology with mucinous epithelial cells or, alternatively, carcinoembryonic antigen fluid levels >192 ng/mL and glucose levels <50 mg/dL.

### Convolutional neural network development

**Figure 1.**
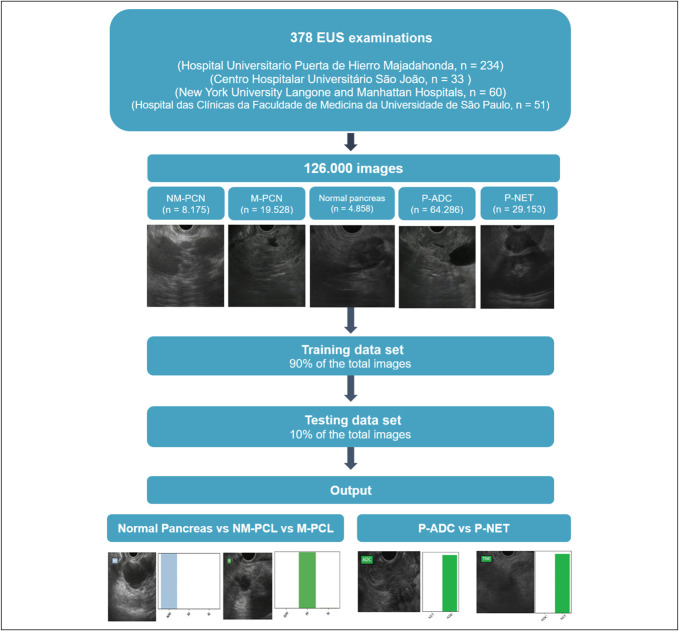
Study design for the development of the convolutional neural network. ADC, adenocarcinoma; M, mucinous; m-PCN, mucinous pancreatic cystic neoplasm; N, normal; NET, neuroendocrine tumor; NM, nonmucinous; NM-PCN, nonmucinous pancreatic cystic neoplasm; P-DAC, pancreatic adenocarcinoma; P-NET, pancreatic neuroendocrine tumor.

A CNN was developed for automatic detection and differentiation of PSL (differentiating P-DAC from P-NET) and PCN (focusing on the distinction between normal pancreatic tissue, M-PCN, and NM-PCN). Figure 1 summarizes the study design flowchart.

A total of 378 patients who underwent EUS were selected for this study. Each patient had a single lesion identified throughout the examination, with a total of 378 lesions. The image data set was compound by both still frames acquired during EUS procedure and images obtained from decomposition of the recorded videos. The EUS videos were fragmented into still images using a VLC media player (VideoLAN, Paris, France). After removal of nonrelevant frames, a total of 126.000 images were used for CNN development. The final data set included 19.528 images of M-PCN, 8.175 NM-PCN, 4.858 images of normal pancreatic tissue, 64.286 P-DAC, and 29.153 P-NET images.

The trinary PCN data set was developed to distinguish between normal pancreatic tissue, NM-PCN, and M-PCN. A total data set of 32.561 images was divided into a training (containing 90% of the total images, n = 29.304 images) and a testing data set (with 10% of the total images, n = 3.257 images), used for evaluation of the models' performance.

On the other hand, the PSL data set focused on the differentiating P-DAC from P-NET. Therefore, 93.439 images were divided similarly to the PCN data set in a training (with 90% of the total images, n = 84.095) and testing data set (with 10% of the total images, n = 9.344 images), used for determining the models' performance.

The *Resnet* model with its weights trained on *ImageNet* was used for creation of the CNN. The convolutional layers of the model were maintained in order to transfer this learning to our data. In addition, the final full connected layers were removed and replaced with new full connected layers according to the number of classes used for classification of EUS images. An initial fully connected layer was included in each of the 2 used block, followed by dropout layers with a drop rate of 0.1. Then, a dense layer with a size dependent on the number of the classification groups was added. Th model had a learning rate of 0.00015, batch size of 128, and a number of epochs of 10. The model was prepared and run in PyTorch. The model's performance analysis was performed with a computer equipped with a 2.1 GHz Intel Xeon Gold 6130 processor (Intel, Santa Clara, CA) and a double NVIDIA Quadro RTX 4000 graphic processing unit (NVIDIA Corporate, Santa Clara, CA).

### Evaluation of the models' performance and statistical analysis

**Figure 2.**
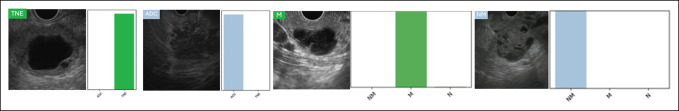
Output obtained from the convolutional neural network. The bars are a representation of the estimated probability by the CNN. The model output was given by the finding with the highest probability. ADC, pancreatic adenocarcinoma; M, mucinous pancreatic cystic neoplasm; N, normal pancreas; NET, pancreatic neuroendocrine tumor; NM, nonmucinous pancreatic cystic neoplasm.

For each image of PCN or PSL, the CNN prediction was associated with a given probability. Indeed, a higher probability revealed a greater confidence in the CNN prediction. The model's output consisted of the predicted classification with the highest probability (Figure 2). The CNN prediction was compared with the current gold standard of histopathological analysis (either by EUS-guided biopsies or cytology or analysis of the surgical specimen). Table 1 translates the confusion matrix between the models' prediction and histopathology analysis (for both PCN and PSL).

**Table 1.** Confusion Matrix for model's prediction vs the histopathological result in the testing data set of the CNN model for both pancreatic cystic lesions and pancreatic solid lesions

	Histopathological result		
	Normal	M-PCN	NM-PCN
CNN prediction			
Normal	479	22	1
M-PCN	7	1,931	5
NM-PCN	0	0	812

	Histopathological result	
	P-DAC	P-NET
CNN prediction		
P-DAC	6,343	477
P-NET	86	2,438

Number of cases.

M-PCN, mucinous pancreatic cystic neoplasm; NM-PCN, nonmucinous pancreatic cystic neoplasm; P-DAC, pancreatic adenocarcinoma; P-NET, pancreatic neuroendocrine tumor.

The model's performance was evaluated through its sensitivity, specificity, positive (PPV) and negative predictive values (NPV), and its accuracy in detecting and distinguishing different PCN and PSL (Table 2). The Sci-kit learn v0.22.2 was used for statistical analysis of the model.

**Table 2.** Performance measures of the testing data set for detection and differentiation of normal pancreas images, pancreatic cystic lesions, and pancreatic solid lesions

	Sn	Sp	PPV	NPV	Acc
Normal pancreas	0.986 (0.970–0.994)	0.992 (0.988–0.995)	0.954 (0.933–0.969	0.998 (0.995–0.999)	0.991 (0.987–0.994)
M-PCN	0.989 (0.983–0.993)	0.991 (0.984–0.995)	0.994 (0.989–0.996)	0.983 (0.975–0.989)	0.990 (0.985–0.993)
NM-PCN	0.993 (0.984–0.997)	0.999 (0.999–1.000)	0.999 (0.996–1.000)	0.998 (0.995–0.999)	0.998 (0.996–0.999)
P-DAC	0.987 (0.984–0.989)	0.837 (0.822–0.850)	0.930 (0.924–0.935)	0.966 (0.958–0.972)	0.940 (0.935–0.944)
P-NET	0.837 (0.822–0.850)	0.987 (0.984–0.989)	0.966 (0.958–0.972)	0.930 (0.924–0.935)	0.940 (0.935–0.944)

(), 95% confidence interval values; Acc, accuracy; M-PCN, mucinous pancreatic cystic neoplasm; NM-PCN, nonmucinous pancreatic cystic neoplasm; NPV, negative predictive value; P-DAC, pancreatic ductal adenocarcinoma; P-NET, pancreatic neuroendocrine tumor; PPV, positive predictive value; Sn, sensitivity; Sp, specificity.

## RESULTS

A total of 378 videos of EUS performed for evaluation of pancreatic lesions during November 2017 and December 2023 in 4 reference centers (Hospital Universitario Puerta de Hierro Majadahonda, n = 234; Centro Hospitalar Universitário São João, n = 33; New York University Langone and Manhattan Hospital, n = 60; Hospital das Clínicas da Faculdade de Medicina da Universidade de São Paulo, n = 51) were used for development of the model. A total of 126.000 frames were extracted for development of CNN: 64.286 of P-DAC, 29.153 of P-NET, 4.858 of the normal pancreas, 19.528 of M-PCN and 8.175 of NM-PCN.

### Pancreatic cystic neoplasms

The trinary CNN was developed to distinguish between normal pancreas images and cystic lesions, differentiating M-PCN from NM-PCN. The deep learning model identified normal pancreas images with 98.6% sensitivity, 99.2% specificity, and 99.1% accuracy. M-PCN were detected with 98.9% sensitivity, 99.1% specificity, a PPV of 99.4%, and an NPV of 98.3%, with a global accuracy of 99%. Finally, the testing data set of our CNN revealed 99.3% sensitivity, 99.9% specificity, 99.9% PPV, 99.8% NPV, and 99.8% accuracy for the diagnosis of NM-PCN.

### Pancreatic solid lesions

The binary CNN was developed to differentiate between the 2 commoner PSL, namely P-DAC and P-NET. The testing data set revealed 94.0% accuracy for the distinction between the lesions, with 98.7% and 83.7% sensitivities for P-DAC and P-NET, respectively.

## DISCUSSION

Pancreatic lesions constitute a diagnostic challenge, with lesion differentiation as a main priority for patient management. EUS, although regarded as the preferred examination in the evaluation of focal pancreatic lesions, suffers from interobserver concordance issues and has suboptimal diagnostic yield. Thus, AI appears as a solution for increasing the diagnostic yield of EUS for pancreatic lesions, increasing biopsy accuracy, and ensuring better patient management. In this multicentric transatlantic study, our group has developed a CNN that excelled in diagnosis and differentiation of both PCN and PSL in EUS images. The CNN had a global accuracy of 94% for distinguishing between P-DAC and P-NET, with an almost perfect discriminatory ability in detecting and distinguishing M-PCN and NM-PCN. The CNN had an image processing time that favors its application in real-time during EUS examinations.

AI application in EUS is a hot topic, with increased evidence that favors the use of AI models to increase examination diagnostic accuracy, guide tissue sampling, or aid in anatomopathological reports (25). Nevertheless, there is still limited evidence in the application of deep learning models for pancreatic lesion differentiation in EUS. Nguon et al focused on the distinction between mucinous cystic neoplasms and serous cystic neoplasms, achieving a global accuracy of nearly 83%, similar to endoscopic visual impression accuracy (26). Despite the promising results, the cited study focused on distinguishing 2 specific types of cystic lesions. Later in 2022, our group developed a CNN for differentiation between M-PCN and NM-PCN in EUS images (23). The CNN revealed 98% accuracy for the distinction between the 2 types of cystic lesions. Indeed, the distinction is relevant because of the risk of malignancy being almost exclusive of M-PCN. In fact, AI could be an important decision aid by predicting the risk of malignancy, therefore, selecting the most appropriate patients for a surgical approach. AI-assisted malignancy risk prediction was also evaluated in a model by Machicado et al, based on 35 histologically confirmed intraductal papillary mucinous neoplasms and images from EUS-guided confocal laser endomicroscopy (27). The developed CNN showed higher diagnostic accuracy than the available International Consensus Guidelines for a surgical approach of these lesions.

Nevertheless, despite the promising results, the distinction between 2 specific types of cystic lesions is insufficient for the development and application of a clinically available AI model. Therefore, the present CNN was not only capable of distinguishing between the 2 subtypes of cystic lesions, but also to differentiate them from normal pancreatic tissue, assuring not only a differentiation ability, but also a detection capacity, fundamental for an accurate identification of cystic lesions, possible leveraging AI-guided EUS biopsies, augmenting their diagnostic accuracy.

Indeed, P-DAC is associated with a poor prognosis because of late diagnosis with unresectable tumor (28). Therefore, EUS is fundamental in the presence of a PSL, to exclude P-DAC or diagnose it in an early stage. AI models have been proposed as a potential transforming factor in assuring an early diagnosis and management of P-DAC (28). In fact, several works have proved AI benefit in diagnosis of P-DAC, either in an image or histopathology level (29,30). Nevertheless, there is a scarcity of works about AI application in EUS for the diagnosis of PSL. A work based on 1490 EUS images from Tonozuka et al revealed that a computer-assisted diagnosis for EUS was accurate in differentiating P-DAC from images of the normal pancreas or chronic pancreatitis, with an area under the curve of 0.94 (31). A study based on 65 EUS examinations showed that a computer-assisted diagnosis was able to accurately differentiate PSL, with a diagnostic accuracy of 97% for P-DAC and 98.5% for P-NET. Despite the promising results, these studies were based on a small number of patients. Our study based on more than 90.000 images of PSL revealed a diagnostic accuracy of 94.0% for distinguishing between the 2 commonest PSL, P-DAC, and P-NET. Given the diagnostic challenge of small P-DACs in patients with chronic pancreatitis, future studies will focus on distinguishing between the commonest PSL (P-DAC and P-NET) and normal pancreas or chronic pancreatitis tissue.

In fact, there is a need to discuss some methodological strengths of the study. First, the gold standard for lesion classification was based on the available histopathology report. Therefore, the diagnosis of a PSL implied a EUS-guided FNA/FNB biopsy or surgical specimen analysis. On the other hand, PCN were diagnosed based on either EUS-guided FNA/FNB analysis, cytology fluid analysis or histopathological analysis of the surgical sample. Indeed, the development of a deep learning model is dependent on a rigorous definition of a gold standard for assuring a correct classification. The absence of a histopathological sample was defined as an exclusion criterion for the CNN data set. Therefore, the strict criteria assured the “ground truth” classification of a PCN or PSL in the CNN development, increasing the quality of the data set.

In addition, it is important to consider the inclusion of images from several available EUS devices, with the development of a CNN that is accurate in different linear echoendoscopes. As a matter of fact, AI implementation in the clinical practice is dependent on the generalization of a technology for use in multiple devices (32). In this context, the findable, accessible, interoperable, reusable guiding principles for data stewardship had postulated interoperability as a main priority in AI technology development and application (33). Therefore, several works address the interoperability challenge, aiming to develop AI models that are useful in clinical practice (34,35). Our group has focused on this concern, using data from different linear EUS devices, partially solving the interoperability challenge. However, our group reckons that the model was trained in a small number of EUS devices. With the development of the model, our group aims to create CNN that is accurate in most EUS devices, amplifying the clinical applicability of the technology.

Last, a significant concern in the development of the deep learning model is the existence of an imbalanced data set, which is not adapted to the population in which the technology will be used. In fact, the demographic bias has been pointed out as a cause of unbalance in the prediction of AI models (36). Indeed, most CNNs are based on a small population sample, limiting the external validity of the results. Our group has focused in minimizing this bias, by including EUS images from 4 reference centers in 2 different continents (Portugal, Spain, the United States, and Brazil). With this methodology, patients from different demographic contexts were included, assuring a more robust CNN that was developed with data from both European and American populations.

Nevertheless, there is a need to consider the limitations of the study. First, albeit being based on 126.000 images from 378 EUS examinations, there was an absence of patient split examination, with the possibility of models' overfitting because of the presence of similar images in both the training and testing data set. In fact, the number of images per examination was variable, ranging between 1 and 7,000 images. Nevertheless, this is a proof-of-concept study, with the first worldwide CNN able to identify and distinguish the main types of both solid and cystic pancreatic lesions in still frames from EUS examinations, with an expected lower technology readiness level, Therefore, future studies will focus on achieving this diagnostic accuracy in a real-time context with the ability of obtaining results in a patient split methodological design.

In addition, this study was developed with a retrospective design. In the future, prospective multicentric studies are necessary to achieve clinical implementation of the models. In addition, the model development was based on still images. Therefore, real-time evaluation of the technology during EUS procedures is also a priority in the future. Finally, our group aims to include data from additional centers and EUS devices, assuring the development of an interoperable technology.

In conclusion, our group developed the first worldwide CNN capable of detection and differentiation of PCN (namely between M-PCN and NM-PCN) and PSL (namely between P-DAC and P-NET), based on a large image data set from 4 reference centers (of both the European and American continent). The development of AI models is of uttermost importance for augmenting EUS diagnostic accuracy, and the availableness of a technology that is efficient in detecting both PCN (differencing them from normal pancreatic tissue) and PSL is a proof of methodological development and robustness. Larger multicentric and prospective studies are needed for the clinical implementation of this technology.

## CONFLICTS OF INTEREST

**Guarantor of the article:** Miguel Mascarenhas Saraiva, MD, PhD.

**Specific author contributions:** M.M. and F.M.: study design, image extraction, drafting of the manuscript, and critical revision of the manuscript. T.R., J.A., M.M., M.J.A., Y.F., A.B., M.F.d.C., B.A., A.C., and F.V.B.: bibliographic review, image extraction, and critical revision of the manuscript. J.F. and J.F.: construction and development of the CNN, statistical analysis, and critical revision of the manuscript. M.G.-H., J.W., T.G., M.E.L., E.H.d.M., S.L., P.M.R., and G.M.: study design and critical manuscript revision. All authors approved the final version of the manuscript.

**Financial support:** The authors recognize NVIDIA support for the graphic unit acquisition.

**Potential competing interests**: None to report.

**Study Highlights**

**WHAT IS KNOWN**

✓ Focal pancreatic lesions have a challenging diagnostic workup, with benign and malign etiologies.
✓ Endoscopic ultrasound (EUS) is typically involved in the diagnostic workup, but its diagnostic yield is suboptimal.

**WHAT IS NEW HERE**

✓ Our group developed the first deep learning model evaluating pleomorphic pancreatic lesions in EUS.
✓ Artificial intelligence algorithms could increase diagnostic yield of EUS for pancreatic lesions.
